# Skeletal fragility in pituitary disease: how can we predict fracture risk?

**DOI:** 10.1007/s11102-024-01447-3

**Published:** 2024-09-06

**Authors:** Fabio Bioletto, Alessandro Maria Berton, Marco Barale, Luigi Simone Aversa, Lorenzo Sauro, Michela Presti, Francesca Mocellini, Noemi Sagone, Ezio Ghigo, Massimo Procopio, Silvia Grottoli

**Affiliations:** 1 Division of Endocrinology, Diabetes and Metabolism, Department of Medical Sciences, University of Turin, Corso Dogliotti 14, Turin, 10126 Italy; 2 Division of Oncological Endocrinology, Department of Medical Sciences, University of Turin, Turin, Italy

**Keywords:** Acromegaly, Cushing’s disease, Hyperprolactinemia, Hypopituitarism, Osteoporosis, Fracture risk

## Abstract

Pituitary hormones play a crucial role in regulating skeletal physiology, and skeletal fragility is a frequent complication of pituitary diseases. The ability to predict the risk of fracture events is crucial for guiding therapeutic decisions; however, in patients with pituitary diseases, fracture risk estimation is particularly challenging. Compared to primary osteoporosis, the evaluation of bone mineral density by dual X-ray absorptiometry is much less informative about fracture risk. Moreover, the reliability of standard fracture risk calculators does not have strong validations in this setting. Morphometric vertebral assessment is currently the cornerstone in the assessment of skeletal fragility in patients with pituitary diseases, as prevalent fractures remain the strongest predictor of future fracture events. In recent years, new tools for evaluating bone quality have shown promising results in assessing bone impairment in patients with pituitary diseases, but most available data are cross-sectional, and evidence regarding the prediction of incident fractures is still scarce. Of note, apart from measures of bone density and bone quality, the estimation of fracture risk in the context of pituitary hyperfunction or hypofunction cannot ignore the evaluation of factors related to the underlying disease, such as its severity and duration, as well as the specific therapies implemented for its treatment. Aim of this review is to provide an up-to-date overview of all major evidence regarding fracture risk prediction in patients with pituitary disease, highlighting the need for a tailored approach that critically integrates all clinical, biochemical, and instrumental data according to the specificities of each disease.

## Introduction

Pituitary hormones play a crucial role in regulating skeletal physiology, and bone fragility is a frequent consequence of pituitary disorders [1]. The clinical relevance of pituitary hormone actions on bone metabolism is demonstrated by the often-severe skeletal damage seen in conditions of hyperfunction or hypofunction of the gland [1].

In the general context of osteoporosis, fragility fractures represent the most important clinical outcome, as they directly reflect the severity of bone damage and are linked to increased disability, morbidity and mortality [2, 3]. Therefore, essentially all clinical actions that are put in place in this area have the ultimate aim to predict fracture risk and, consequently, to implement timely therapeutic actions able to reduce the probability of fracture events in primary or secondary prevention [2, 3].

In the setting of primary osteoporosis, several factors have been identified as predictors of an individual’s fracture risk [4, 5]. Over the years, these factors have also been combined into various integrated predictive tools, that have been developed and extensively validated for fracture risk prediction [6–8]. Among these, the one that is most widely used is FRAX^®^, which combines several clinical risk factors with bone mineral density (BMD) to calculate the 10-year probability of hip fracture and the 10-year probability of a major osteoporotic fracture (clinical spine, forearm, hip or shoulder fracture) [6].

In the setting of secondary osteoporosis, obtaining a reliable estimate of fracture risk can be much less straightforward [1, 9]. Some fracture risk calculators, including FRAX^®^ itself, provide the possibility of reporting the presence of a condition causing secondary osteoporosis [6]; however, this is included in the calculator as a non-specific information, without the ability to differentiate between different causes of secondary osteoporosis, nor to further stratify the assessment of fracture risk according to any additional parameter, such as disease duration and/or severity.

Moreover, the reliability of these risk calculators has been much less extensively validated in the contexts of secondary osteoporosis, and their generalizability is not obvious, given the different pathophysiology of bone damage in each disease [1, 9]. The classic predictors used in FRAX^®^ are in fact often much less informative in the context of secondary osteoporosis [1, 9]. The clearest example of this is probably represented by the role of BMD: indeed, while accounting for more than two-thirds of the whole bone strength in primary osteoporosis, in the contexts of secondary osteoporosis its measurement is much less informative about fracture risk, if at all [1, 9]. In fact, bone fragility in secondary osteoporosis is much more significantly influenced by alterations in bone quality and microarchitecture, which are not captured by BMD [1, 9, 10].

In recent years, many efforts have been made to overcome this limitation by introducing techniques capable to noninvasively assess bone quality, such as trabecular bone score (TBS) [10–12] and high-resolution peripheral quantitative computed tomography (HR-pQCT) [13, 14], among others. These parameters have been evaluated in several forms of secondary osteoporosis [15–21], often with promising results. However, given the relative rarity of these conditions, the sample sizes of these studies cannot match those available for primary osteoporosis, and the lack of adequate follow-up is often a significant limitation. Additionally, bone turnover markers have emerged as well as important tools in evaluating bone metabolism and turnover, providing further insights into fracture risk assessment [22, 23].

The aim of this review is to address the clinical question of fracture risk prediction in patients with pituitary disease, evaluating the available evidence about predictors of fragility fractures in each context of hyperfunction or hypofunction of the gland.

### Acromegaly

Traditionally, growth hormone (GH) and insulin-like growth factor 1 (IGF-1) were viewed as anabolic hormones that benefit the skeleton by promoting longitudinal bone growth, bone modeling, and remodeling [24]. However, in the last two decades, compelling evidence showed that a pathological GH excess actually contributes to skeletal fragility, sustained by increased bone turnover and abnormalities in bone microstructure, ultimately raising fracture risk [25–27]. Fractures are frequently asymptomatic and predominantly involve the thoracic spine, with anterior wedge deformities leading to kyphosis [28].

The risk of fractures is tightly related to the exposure to elevated GH and IGF-1 levels. The prevalence of vertebral fractures at diagnosis has been shown to be correlated with a longer estimated diagnostic delay [29] and with higher random serum GH levels [30]. Moreover, during the course of the disease, patients with active acromegaly are characterized by a markedly higher fracture risk compared to those with a biochemically controlled disease [27, 31]. Of note, an effect related specifically to the individual drugs used for the medical treatment of acromegaly may also be present, with some evidence supporting a lower fracture risk in patients with biochemically active disease treated with pasireotide compared to those treated with pegvisomant [32].

Acromegaly is not characterized by a significant alteration of BMD [26], with most patients showing normal or even increased bone density at various skeletal sites, in large part due to different distribution of trabecular and cortical bones, concomitant joint degenerative disorders characterized by osteophytes and facet joint hypertrophy, increased periosteal ossification and bone enlargement [1]. In line with these findings, BMD does not represent a reliable predictor of fractures in the setting of acromegaly; in fact, studies comparing acromegalic patients with and without VFs did not find any significant difference in BMD between the two groups, and VFs occurred even in patients with normal bone density [26, 33].

Currently, new tools are being studied to assess bone quality and predict fracture risk in acromegalic patients. The TBS, for example, has been found to be lower in acromegalic patients than in non-acromegalic controls, and was significantly lower in acromegalic patients with prevalent VFs than in those without [15]. Similar data of impaired bone quality can be captured by HR-pQCT, with studies showing an alteration of both cortical and trabecular microarchitectural parameters [34, 35]. Bone microindentation, a micro-invasive method that measures bone material strength by a probe on the tibial surface, showed lower values in acromegaly patients even after biochemical control, thus suggesting persistence – at least to a certain degree, of cortical bone alterations [36]. Other indices and techniques, such as quantitative ultrasound (QUS) and 3D-SHAPER, have also been investigated as measures of skeletal health in acromegalic patients [37–39]. Overall, however, up to date there is no clear longitudinal data demonstrating a role of these indices as reliable predictors of incident fractures in this setting.

As in primary osteoporosis, a strong relationship between prevalent and incident VFs has been demonstrated also in acromegalic patients [40, 41]. In light of this, morphometric assessment of VFs even in the absence of specific symptoms and signs has to be considered as the cornerstone in the assessment of skeletal fragility in all patients with acromegaly, with an initial evaluation at diagnosis and subsequent regular monitoring during follow-up [30, 42], at intervals tailored to each subject’s evolving clinical condition, with special attention to male patients, patients with untreated hypogonadism, history of non-traumatic VFs, decrease in BMD, kyphosis, and biochemically active disease [26, 30, 41]. Of note, similar again to primary osteoporosis, fracture risk is also negatively associated with vitamin 25(OH)D levels, and appears to be reduced by cholecalciferol supplementation [43].

In terms of bone turnover markers, conventional and novel markers such as serum osteocalcin, bone-specific alkaline phosphatase (BALP), C-terminal telopeptide of type I collagen (CTX), procollagen type 1 N-terminal propeptide (P1NP) and sclerostin have been investigated to assess bone turnover in acromegaly [33, 44–46]. Elevated levels of these markers may correlate with an increased skeletal fragility and may serve as additional tools for evaluating bone health in these patients, although their exact role in predicting fracture risk requires further longitudinal studies.

### Cushing’s disease

Endogenous hypercortisolism is an established cause of osteoporosis and bone damage, with fragility fractures representing in some cases the presenting clinical feature of the disease [47, 48]. From a pathophysiological point of view, glucocorticoid-induced osteoporosis is determined mainly by decreased bone formation, and to some extent by a slight initial increase in bone resorption, with a greater impact on trabecular compared to cortical bone [49].

Literature data indicate that fractures caused by glucocorticoid excess may represent an early event during the course of the disease [50–52], but their occurrence then increases with the duration of hypercortisolism [53]. The risk of fractures is also related to the severity of glucocorticoid excess, with a direct correlation with 24 h urinary free cortisol even after adjustment for multiple confounders [53, 54]. Interestingly, vertebral and rib fractures were significantly more common in men than in women in some research, but this difference could possibly be related to sex-specific differences in disease activity [54, 55]. Of note, fracture risk significantly decreases after adequate cure or treatment, together with an improvement in bone mineralization [51, 56, 57].

Differently from acromegaly, the measurement of BMD by DXA retains an independent prognostic role for the prediction of fracture risk in patients with endogenous hypercortisolism [55, 58, 59]. A decrease in BMD was consistently reported in patients with Cushing’s syndrome, with an increase in the prevalence of osteopenia and osteoporosis compared to controls [53, 60]; more specifically, in agreement with the notion that trabecular bone is more impacted by glucocorticoids than cortical bone, lumbar spine BMD tends to be impacted more than the hip and the forearm [49]. Moreover, several studies demonstrated a correlation between BMD reduction and fracture risk, with BMD values being a significant predictor of fractures both in treated and in untreated patients [55, 58–60].

Notably, however, the detrimental effects of glucocorticoid excess on bone health are not limited to a loss of bone mass [47, 48]. Patients with Cushing’s syndrome may often present fragility fractures even with mild or no reduction in BMD, and the increase in fracture risk overall exceeds the one that could be expected based on the reduction of BMD alone [53–55, 59]. In recent years, therefore, several studies have tried to fill this gap by evaluating indices of bone quality. TBS has been shown to be significantly lower in patients with Cushing’s syndrome than in controls [61], and lower TBS values have been generally reported in Cushing’s disease patients with prevalent VFs than in those without [20, 42, 61, 62]. Useful information can also be provided by HR-pQCT, which demonstrated lower cortical thickness, lower cortical area as well as lower total and cortical density in patients with Cushing’s syndrome compared to controls, even after the exclusion of hypogonadal individuals [63].

As in primary osteoporosis, vertebral morphometry helps in recognizing patients with prevalent VFs, which indicate skeletal fragility and an increased likelihood of further future fracture events [64]. The morphometric assessment appears to be currently the most effective screening approach for patients with Cushing’s syndrome, with an initial evaluation to be performed at diagnosis and subsequent regular monitoring during follow-up [50–53]; in this regard, a novel approach combining DXA and vertebral morphometry has been recently proposed for the management of bone comorbidity in this setting [64].

Finally, in terms of bone turnover markers, their assessment has provided valuable insights into the bone remodeling processes in patients with Cushing’s disease. Markers of bone formation, such as serum osteocalcin and P1NP, are typically suppressed due to the inhibitory effects of glucocorticoids on osteoblast function [49, 65–67]; conversely, markers of bone resorption, such as CTX, may be either normal or slightly elevated [49, 65, 67]. Nevertheless, although these markers offer additional information on the underlying bone dynamics in Cushing’s disease, clear evidence of an independent prognostic role of these indices in the prediction of incident fracture risk is lacking.

### Hyperprolactinemia and hypogonadism

Bone derangement in patients with hypogonadism is mostly due to the loss of the skeletal protective effects exerted by sex hormones [1]. In fact, both estrogens and androgens attenuate osteoblast and osteocyte apoptosis and suppress bone resorption [1]. The functional suppression of the hypothalamus-pituitary-gonadal axis also represents the main mediator of bone derangement in patients with hyperprolactinemia, although there is some evidence suggesting that prolactin excess could also exert direct effects on bone cells [68].

Few studies investigated fracture risk in patients with prolactinoma. Vestergaard et al. reported a 1.6-fold increase in prevalence of clinical fractures in newly diagnosed prolactinoma as compared with controls [69], while two cross-sectional studies depicted a higher prevalence of radiological VFs both in women and men with prolactinoma (32.6% and 37.5%, respectively) when compared to controls [70, 71]. Accordingly, a recent meta-analysis confirmed that patients with prolactinoma had a higher prevalence of fragility fractures than controls [72]. In these studies, lower BMD values, a longer duration of disease, and hyperprolactinemia per se, regardless of gonadal status, were significantly related to VFs [70, 71], while treatment with cabergoline was associated with a lower risk of VFs [73] and improved BMD [74–78].

With respect to BMD, several studies investigated the prevalence of reduced bone density in patients with prolactinoma. A study evaluating men with prolactinoma naïve to treatment showed a higher prevalence of osteopenia or osteoporosis compared to controls, particularly at the lumbar spine, thus suggesting that trabecular bone was damaged earlier than cortical bone [74]. In some studies, BMD was found to be correlated with prolactin levels and duration of the disease [74, 79]. In an observational cohort study, Andereggen et al. found that persistent hyperprolactinemia and male sex were independent risk factors for long-term bone mass loss, while treatment with dopamine agonists showed no effects on BMD [80]. Other authors reported a significant association between BMD values and hypogonadism in patients with prolactinoma; specifically, bone loss was associated with the duration of amenorrhea in premenopausal women, and was not completely restored despite subsequent normalization of prolactin levels in adolescence-onset patients with prolactinoma, suggesting that bone loss was mainly associated with gonadal insufficiency rather than with prolactin excess [42]. The relationship between BMD and fractures has been investigated by fewer studies; interestingly, prolactinoma patients with fractures showed lower lumbar spine BMD as compared with patients without fractures, thus suggesting a possible role of bone density in the stratification of fracture risk [70, 71].

Apart from bone density, some evidence exists suggesting an impaired bone quality in patients with prolactinoma; one study, evaluating bone microarchitecture by HR-pQCT, found a lower trabecular and cortical thickness at both radius and tibia in patients with prolactinoma with respect to controls [81]; higher circulating prolactin levels and male sex were associated with the severity of bone damage [81].

Apart from patients with prolactinomas, a higher risk of osteoporosis also connotes patients with idiopathic hypogonadotropic hypogonadism, mainly due to the achievement of a lower peak of bone mass in early adulthood [82], with a significant improvement after hormone replacement therapy [83], suggesting that an early diagnosis and a good compliance to hormonal replacement therapy are crucial for bone health [84]. However, several studies reported that BMD and bone microarchitecture still remained partially impaired in these patients despite long-term hormonal treatment [85–87]. Notably, the association with fragility fractures has been mostly investigated in primary rather than secondary hypogonadism, so clear data are lacking in this latter clinical setting.

### Growth hormone deficiency

GH and IGF-1 exert a pivotal role in bone remodelling [88], through both direct skeletal effects and indirect mechanisms, such as those mediated by the interplay with the activity of renal 1 alpha-hydroxylase and thus to the metabolism of vitamin D [89]. Overall, patients with GH deficiency (GHD) are characterized by a marked reduction in bone turnover, with a suppression of both bone resorption and bone formation [90–92]. In adult patients with GHD, the risk of fractures is two to five times higher compared to control subjects, independent of the presence of other pituitary hormone deficiencies [1, 93–97]. Initial studies reported that this increase in fracture risk mostly involved cortical bone sites [94–96]; in the following years, however, the involvement of trabecular bone was demonstrated as well [93].

Literature data indicate that fractures in this setting have comparable prevalence in males and females, thus suggesting skeletal fragility as a complication of GHD independently from sex [93]. The risk of incident fractures progresses with the duration of the disease [98]. However, it seems to be attenuated in patients receiving GH replacement, who show a lower incidence rate of fractures compared to those untreated, even after adjustment for multiple confounders [24, 98–100].

With respect to BMD, patients with GHD are characterized by lower BMD values compared to controls [101]. Risk factors for lower bone density are represented by low body mass index (BMI), disease duration, disease severity, age of onset, and other concomitant hormonal derangement [102]. With respect to the age of onset, a clear dichotomy arises between childhood-onset GHD and adult-onset GHD, with the former being characterized by lower BMD values compared to the latter, likely due to the defective achievement of peak bone mass [103–107]. Nevertheless, age of onset represents a significant predictor of bone loss even when restricting the evaluation to adult-onset GHD, with patients in their early adulthood presenting with a more severe bone impairment compared to controls, while those with an age of onset above 55 years often do not differ significantly from non-GHD counterparts [99, 101, 108–110]. Notably, a significant association between BMD values and VFs has been shown, thus suggesting that the measurement of BMD by DXA retains an independent prognostic role in the estimation of fracture risk [93, 98]. However, fractures in patients with GHD can occur in a significant percentage also in patients with normal BMD [93].

A possible explanation for this would be an alteration of bone microarchitecture, not captured by BMD. However, data about bone microarchitectural quality in patients with GHD are relatively limited and overall inconclusive. The analysis of the bioptical specimens obtained by Bravenboer did not permit to highlight an altered trabecular structure in GHD males, nor a significant difference on the same patients before and after 5 years of GH replacement therapy [111]. Data derived from HR-pQCT are also relatively scarce; overall they suggest an impairment of bone quality in patients with childhood-onset GHD [112], while no differences were found in the setting of adult-onset GHD [35]. A few studies have evaluated TBS in patients with GHD; though limited by the relatively low sample size, no remarkable impairment of TBS was noted, with mean TBS values being in the normal range in most cases [113–115]. Of note, however, interesting results have been observed when evaluating the response of TBS to GH replacement therapy: in fact, in a study evaluating the effect of 2 years of GH replacement in 57 patients with adult-onset GHD, a significant increase of TBS was observed in patients with vitamin 25(OH)D levels above the 50th percentile, while a decrease was observed in those below [113, 116]; this finding further supports the relevance of the aforementioned interplay between somatotroph axis activity and vitamin D metabolism, possibly suggesting that sufficient levels of vitamin 25(OH)D are required to enhance the skeletal benefits of IGF-1-mediated stimulation of renal 1 alpha-hydroxylase.

Notably, also in the context of GHD, the presence of prevalent VFs is a very strong indicator of skeletal fragility and incident fracture risk [98]. In light of this, the morphometric assessment of VFs, even in the absence of specific signs or symptoms, represents an essential evaluation tool to adequately stratify the risk of future fracture events [1, 98].

### Central adrenal insufficiency

As already discussed for Cushing’s syndrome, glucocorticoids play a key role in the regulation of bone metabolism [49]. In the setting of hypopituitarism, clinical data indicate that the link between central adrenal insufficiency and detrimental bone outcomes is primarily due to the potential negative effects of excessive hormone replacement [117]. An overtreatment of central hypoadrenalism, in fact, is relatively common, both because of the lack of reliable biomarkers of hormonal actions and due to the fact that replacement therapy does not completely mirror the endogenous cortisol production [117].

Despite these premises, however, data on fractures in central hypoadrenalism are scarce. Some studies did not show any increased fracture risk in hypopituitarism patients treated with glucocorticoid replacement therapy, but the analysis was limited to clinical fractures and was not stratified by glucocorticoid replacement dose [95, 102]. On the other hand, when accounting for glucocorticoid dose, a higher prevalence of radiological VFs was found in patients treated with a hydrocortisone dose > 28 mg/day, mainly when GHD was not replaced [118], consistent with the pathophysiological notion of an increased peripheral tissue exposure to glucocorticoids when GH is deficient [118–120]. Overall, this is also in line with the general knowledge of a dose-response relationship between glucocorticoid replacement and bone damage, as demonstrated by data on bone turnover markers and BMD both in the setting of primary and secondary (central) adrenal insufficiency [121–124].

Interestingly, apart from the glucocorticoid dose, the specific glucocorticoid formulation may also play a role; in recent years, in fact, some evidence emerged about possible advantages on bone outcomes of dual-release hydrocortisone formulations compared to conventional ones [16, 125–127].

### Central hypothyroidism

Both thyroid hormones and thyroid-stimulating hormone (TSH) play a relevant role in the regulation of bone remodeling and mineralization [128–131]. Thyroid hormones exert catabolic effects in adult bone, and their excess can cause bone loss by increasing bone resorption [128, 129, 132]. Conversely, an association between VFs and lower fT3 levels has also been recently observed in a cohort of patients with non-functioning pituitary adenoma (NFPA), thus suggesting that secondary hypothyroidism may play a role as well [133]. There is also evidence that TSH may have an independent osteoprotective effect by inhibiting osteoclastogenesis and bone resorption, although this appears to be overcome by the effects of thyroid hormones in most clinical settings [130, 131, 134, 135].

Nevertheless, despite pathophysiological knowledge suggests that central hypothyroidism could independently contribute to skeletal fragility because of the detrimental impacts of low TSH levels on bone remodeling, clinical studies suggest that the association between central hypothyroidism and adverse bone outcomes mostly reflects the potential negative effects of hormone over-replacement, which may more easily occur in patients with central hypothyroidism due to the impossibility to use TSH as a monitoring parameter [136, 137].

The detrimental effect of excessive thyroid hormone replacement on bone density and fractures has been widely demonstrated in the setting of primary hypothyroidism [138, 139]. Specific evidence in the setting of central hypothyroidism is relatively scarce, but overall agrees with that found in primary hypothyroidism. In particular, Mazziotti et al. showed that higher daily doses of L-T4 are associated with radiological VFs in patients with hypopituitarism [140]. A high prevalence of fractures was specifically found in patients receiving L-T4 doses > 1.3 µg/kg/day [140]. Moreover, a significant correlation between higher serum fT4 levels and VFs was also found [140].

### AVP and oxytocin deficiency

Clinical evidence regarding bone health in patients with arginine vasopressin (AVP) and/or oxytocin deficiency is relatively limited. From a pathophysiological perspective, receptors for both AVP and oxytocin are present in osteoblasts and osteoclasts, apparently exerting opposite actions in preclinical studies [141]; overall, AVP is attributed with an overall negative regulatory role on bone metabolism, reducing bone formation while concurrently enhancing bone resorption [141, 142]; in contrast, oxytocin overall exhibits an anabolic effect by promoting osteoblast differentiation and function [143]. Despite this pathophysiological evidence, however, data about the fracture risk in patients with AVP and/or oxytocin deficiency are scarce.

An increased risk of fracture in male patients was observed in a large retrospective cohort of hypopituitary patients also affected by AVP deficiency [95]; in this regard, the authors attributed this finding to a more extensive pituitary damage, as well as a possible visual deficit, leading to a greater risk of falls. Literature data also indicate a prominent effect of sodium concentration on bone [141, 144, 145]. In this context, the possible condition of prolonged hyponatremia, which is the most common side effect of overtreatment with desmopressin, particularly in elderly patients with age-associated reduction in renal function, may represents a consistent risk factor for osteoporosis and fragility fractures more than AVP deficiency itself [141, 146]. With respect to bone density, a cross-sectional cohort study by Pivonello et al. reported a reduction in BMD in the lumbar spine and femoral neck, associated with low levels of serum osteocalcin, in young patients with AVP deficiency compared to healthy subjects of the same sex and age [147]. Of note, no significantly higher BMD value was observed in those patients receiving chronic replacement treatment with intranasal desmopressin.

With regard to oxytocin, data in humans are even scarcer. A positive correlation between fasting oxytocin levels and BMD has been described in both pre and postmenopausal women, as well as in hypopituitary men, but so far no clear correlation has been reported between oxytocin values and osteoporotic fractures [148]. Furthermore, oxytocin released from the neurohypophysis and stimulated by childbirth and breastfeeding, appears to have minimal impact on bone metabolism, while peripheral oxytocin is synthesized mainly by adipocytes, gonads, uterus and also osteoblasts, under estrogen stimulation [149].

## Conclusions

**Fig. 1:**
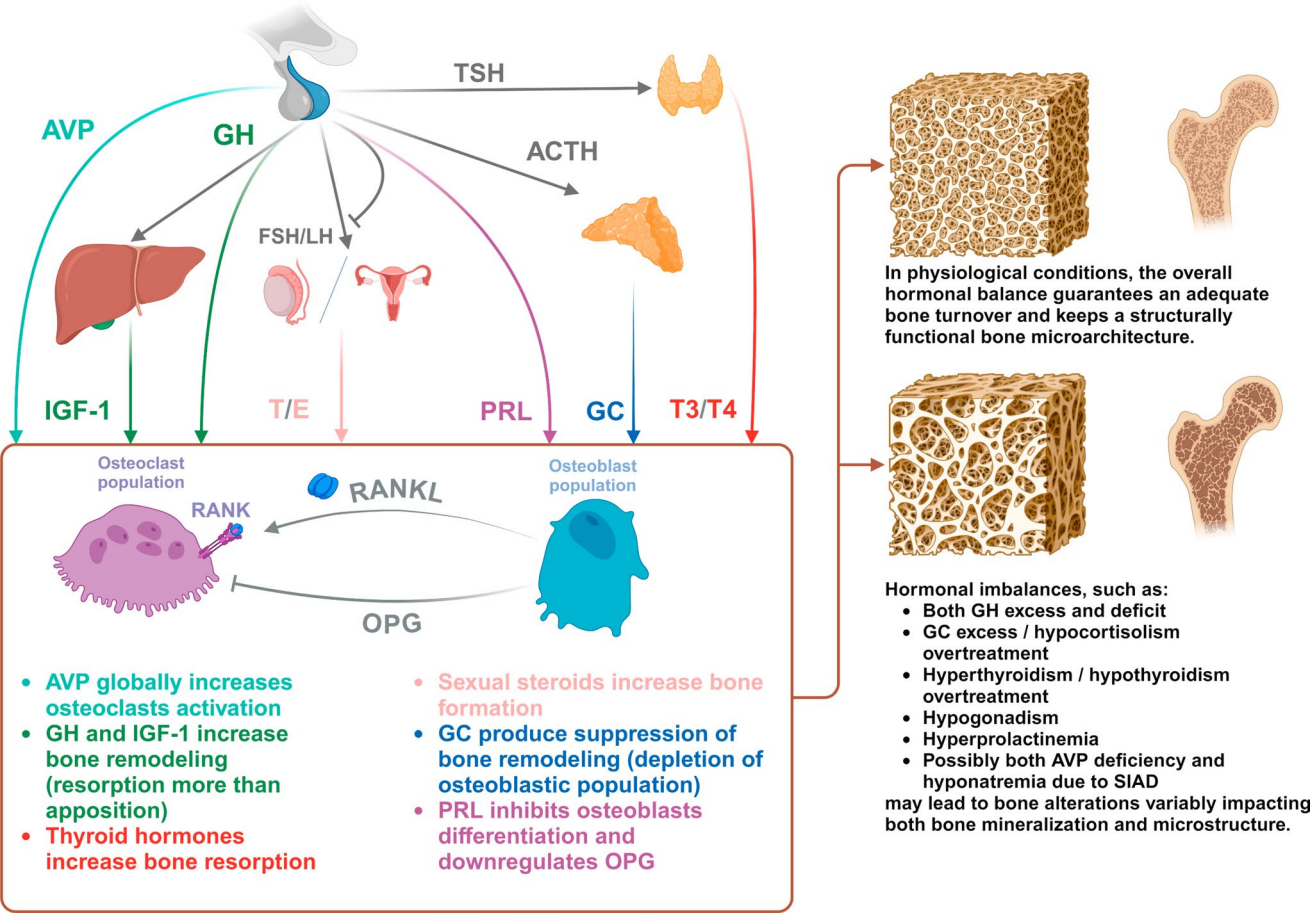
Main effects of pituitary hormones and their peripheral effectors on bone structure and metabolism in adulthood. Abbreviations: ACTH, Adrenocorticotropic Hormone; AVP, Arginine-Vasopressin; E, Estrogens; FSH, Follicle-Stimulating Hormone; GC, Glucocorticoids; GH, Growth Hormone; IGF-1, Insulin-like Growth Factor 1; LH, Luteinizing Hormone; OPG, Osteoprotegerin; PRL, Prolactin; RANK, Receptor Activator of the Nuclear Factor κB; RANKL, RANK-ligand; T, Testosterone; T3, Triiodothyronine; T4, Thyroxine; TSH, Thyroid Stimulating Hormone

Pituitary hormones are essential for the regulation of skeletal physiology, with bone fragility often being a common outcome of pituitary disorders (Fig. 1). The management of skeletal fragility in patients with pituitary disease presents several challenges, as the bone damage associated with these conditions is often caused by mechanisms that differ significantly from those characterizing primary osteoporosis. The ability to predict the risk of fracture events is crucial for guiding therapeutic decisions; however, the reliability of standard fracture risk assessment tools in this context is not obvious. Their use appears not unreasonable to obtain a rough estimate of fracture risk, but the final clinical assessment must first acknowledge the limitations of the parameters used, and subsequently integrate many relevant additional information that are specific to each disease context.

As discussed, the assessment of BMD plays a less significant role in patients with pituitary disease. In recent years, new tools for evaluating bone quality have shown promising results in assessing bone impairment in this setting, but it must be acknowledged that most available data are cross-sectional, and evidence regarding the prediction of incident fractures is overall scarce. On the other hand, morphometric vertebral assessment has substantial evidence supporting its ability to predict incident fractures also in this setting, and currently remains the mainstay for the evaluation of skeletal status in pituitary diseases. Finally, but not less importantly, the estimation of fracture risk in the context of pituitary hyperfunction or hypofunction cannot ignore the evaluation of factors related to the underlying disease, such as its severity and duration, as well as the specific therapies implemented for its treatment (Table 1).

**Table 1:** Descriptive summary of the main factors that can be considered to predict fracture risk in different contexts of pituitary disease. Data for which the available evidence is relatively limited are given in parentheses. Of note, more than one hormonal alteration can potentially coexist in the same patient, and all should be considered in the estimation of fracture risk

Pituitary disease	BMD	Measures of bone quality	Prevalent fractures	Other
Acromegaly	No	(Yes)	Yes	• Disease duration (including diagnostic delay) • Disease control (uncontrolled vs. controlled) • (Male sex)
Cushing’s disease	Yes	(Yes)	Yes	• Disease duration • Disease activity (24 h-UFC) • (Male sex)
Hyperprolactinemia and hypogonadism	Yes	(Yes)	Yes	• Disease duration • Disease activity (s-PRL) • (Male sex)
GH deficiency	Yes	Unclear	Yes	• Age of onset • Disease duration • Treatment approach (untreated vs. treated)
Central adrenal insufficiency	Yes	Unclear	Yes	• Glucocorticoid daily dose • (Glucocorticoid formulation)
Central hypothyroidism	Yes	Unclear	Yes	• L-T4 daily dose
AVP and/or oxytocin deficiency	(Yes)	Unclear	Yes	• (Hyponatremia deriving from desmopressin overtreatment)

*Abbreviations* 24 h-UFC, 24 h urinary free cortisol; AVP, arginine-vasopressin; BMD, bone mineral density; GH, growth hormone; HR-pQCT, high-resolution peripheral quantitative computed tomography; L-T4, levothyroxine; s-PRL, serum prolactin; rhGH, recombinant human growth hormone; TBS, trabecular bone score

In conclusion, therefore, the assessment of fracture risk in patients with pituitary disease cannot be solely performed by using the algorithms used in primary osteoporosis. Instead, it must involve a more comprehensive evaluation that considers the specificities of each condition, critically integrating all clinical, biochemical, and instrumental data according to their specific ability to predict fracture risk in each disease. Given the complexity of factors at play, referral to multidisciplinary pituitary tumor centers of excellence appears to be of key importance in improving outcomes.

## Data Availability

No datasets were generated or analysed during the current study.
